# Synergistic Hydrolysis of Soy Proteins Using Immobilized Proteases: Assessing Peptide Profiles

**DOI:** 10.3390/foods12224115

**Published:** 2023-11-13

**Authors:** Yuhong Mao, Lan Chen, Luyan Zhang, Yangyang Bian, Chun Meng

**Affiliations:** 1 Fujian Key Laboratory of Marine Enzyme Engineering, College of Biological Science and Technology, Fuzhou University, Fuzhou 350116, China; lannarychen@163.com (L.C.); luyan990113@163.com (L.Z.); mengchun@fzu.edu.cn (C.M.); 2 The College of Life Science, Northwest University, Xi’an 710069, China; bianyy@nwu.edu.cn

**Keywords:** Alcalase, Flavorzyme, immobilization, soy proteins, peptidomics, bitterness, bioactivity

## Abstract

Because of the health benefits and economic opportunities, extracting bioactive peptides from plant proteins, often food processing by-products, garners significant interest. However, the high enzyme costs and the emergence of bitter peptides have posed significant challenges in production. This study achieved the immobilization of Alcalase and Flavorzyme using cost-effective SiO_2_ microparticles. Mussel-inspired chemistry and biocompatible polymers were employed, with genipin replacing glutaraldehyde for safer crosslinking. This approach yielded an enzyme loading capacity of approximately 25 mg/g support, with specific activity levels reaching around 180 U/mg for immobilized Alcalase (IA) and 35 U/mg for immobilized Flavorzyme (IF). These immobilized proteases exhibited improved activity and stability across a broader pH and temperature range. During the hydrolysis of soy proteins, the use of immobilized proteases avoided the thermal inactivation step, resulting in fewer peptide aggregates. Moreover, this study applied peptidomics and bioinformatics to profile peptides in each hydrolysate and identify bioactive ones. Cascade hydrolysis with IA and IF reduced the presence of bitter peptides by approximately 20%. Additionally, 50% of the identified peptides were predicted to have bioactive properties after in silico digestion simulation. This work offers a cost-effective way of generating bioactive peptides from soy proteins with reducing potential bitterness.

## 1. Introduction

Peptides, commonly consisting of fewer than 50 amino acids, play crucial roles in various biological processes. Interest in bioactive peptides research is continuously surging because of their capacity to benefit health. These peptides exhibit diverse bioactivities, such as antioxidative, antihypertensive, and antimicrobial properties [1,2]. Bioactive peptides can be potentially derived from various proteins, particularly those generated as by-products during food processing, which presents an increasingly promising approach to reduce waste while recovering valuable compounds. Plants, such as legumes and cereals, are widely consumed, and their agro-industrial remnants provide an economically viable and environmentally sustainable source of proteins. In fact, the health-promoting and disease-preventing properties of peptides obtained from various plant proteins are gaining increased attention [1,3,4]. However, the production of bioactive peptides from hydrolyzing plant proteins for food applications still remains challenging [3,4,5]. Specifically, obtaining the desired bioactive peptides demands meticulous control of enzymatic hydrolysis, with the added complexity of ensuring consistent quality across batches [4]. Striking a delicate equilibrium between bioactive peptide generation and preserving the intended sensory characteristics of the food is another hurdle [4,5]. Ensuring that these products possess both health benefits and an acceptable taste and appearance is pivotal for market acceptance. Moreover, the cost of producing bioactive peptides through enzymatic hydrolysis can be substantial due to the relatively high enzyme prices, while cost-effectiveness is a significant consideration in food production.

To address cost and process control issues, using immobilized proteases offers a feasible solution [6]. Immobilization can enhance the stability of proteases, making them more resilient to a wider range of pH and temperature conditions, which is particularly beneficial for hydrolyzing less-soluble plant proteins. The reusability of immobilized proteases cuts production costs, and their easy separation from the reaction mixture reduces the risk of enzyme contamination in the final product, eliminating the need for an inactivation step that could lead to hydrophobic peptide precipitation [7]. Immobilized enzymes can be employed in various reactor setups, providing production flexibility and process design options. Consequently, the application of immobilized proteases can provide more precise control over the hydrolysis process, resulting in consistent product quality across batches. However, the initial cost of immobilization, including support materials and equipment, can be higher compared to working with free enzymes [6]. In addition, immobilization processes can sometimes lead to a loss of enzyme activity due to conformational changes or steric hindrance, reducing the overall catalytic efficiency of the enzyme [6]. Therefore, achieving a cost-effective immobilization and retaining the maximum activity of immobilized enzymes still remain a challenge.

When it comes to preserving the desired sensory characteristics of bioactive peptides, the choice of enzymes plays a crucial role. Numerous studies [4,8,9,10] have demonstrated that Alcalase, an endopeptidase derived from *Bacillus licheniformis* [9,10,11], stands out as one of the most effective proteases for breaking down plant proteins to produce bioactive peptides. Nevertheless, a common issue associated with the hydrolysis of plant proteins using Alcalase is the development of bitterness. This bitterness arises from the exposure of hydrophobic amino acids, which are abundant in most plant proteins. When these hydrophobic amino acids are present in specific peptide sequences, they can trigger a bitter taste sensation [12,13]. To potentially alleviate this bitterness, the strategy involves using exo-peptidases like Flavorzyme to cleave the exposed hydrophobic amino acids from the C-termini and N-termini of peptides with a bitter taste [12]. Compared with other strategies, such as removing the bitter peptides or concealing their bitter flavor, further hydrolysis with exo-peptidases offers greater nutritional and economic advantages [14].

Following the above strategies, this study first achieved cost-effective immobilization of proteases, i.e., Alcalase and Flavorzyme, utilizing readily available and cheap SiO_2_ microparticles as a support. With mussel-inspired chemistry [15], this bare SiO_2_ particle was initially modified with biocompatible polymers, i.e., polyethylenimine (PEI), self-polymerized polydopamine (PDA), or a combination of both. Alcalase and Flavorzyme were individually immobilized on the functionalized SiO_2_ particles via covalent crosslinking using bifunctional reagents. Although various enzymes [6,15,16,17,18] have been reported to be successfully immobilized on the materials coated with PEI or PDA, how their characteristics, such as molecular weight (MW) and functional group, affect enzyme performance has seldom been discussed. Moreover, glutaraldehyde (GA) is perhaps the most utilized bifunctional reagent for enzyme immobilization, while it is toxic and its induced reactions are relatively quick and hard to control [19]. Genipin (GP), obtained from the extract of *Gardenia jasminoides* fruit, is a reagent gaining popularity due to its excellent crosslinking properties and low toxicity [20], making this compound an ideal alterative to GA. Therefore, this study focused on improving the performance of immobilized proteases by comparing coating polymers as well as the bifunctional reagents.

Secondly, these immobilized proteases were employed in a synergistic process to hydrolyze soy proteins, a typical plant protein mixture known for producing various bioactive peptides while often accompanied by a bitter taste issue [14]. The study compared the efficiency of these immobilized proteases across different hydrolysis processes, as well as against the performance of free protease counterparts. Lastly, we utilized label-free quantification (LFQ) peptidomics analysis to profile the complete set of peptides present in the hydrolysates obtained from various processes. Subsequently, bioinformatic tools were applied to predict the bioactivities of each identified peptide following in silico digestion with digestive enzymes, as well as to assess their initial bitterness.

## 2. Materials and Methods

### 2.1. Materials and Chemicals

Silicon dioxide (SiO_2_) microparticles (10 μm in diameter) were obtained from Aladdin Biochemical Technology Co., Ltd. (Shanghai, China). Flavorzyme and Alcalase 2.4 L were purchased from Yuanye Bio-Technology Co., Ltd. (Shanghai, China) and Novozymes China Biotechnology Co., Ltd. (Beijing, China), respectively. Polyethylenimine (PEI) with molecular weights of 2000 Da (PEI-L) and 750,000 Da (PEI-H) was a gift from BASF China Co., Ltd. (Shanghai, China). Genipin (GP) was purchased from Zhixin Co., Ltd. (Linchuan, China). Soy protein isolate (SPI) was donated by Wonderful Industrial Group Co., Ltd. (Dezhou, China). Casein, glutaraldehyde (GA), Folin and Ciocalteu’s phenol reagent, dopamine (DA), bicinchoninic acid (BCA) protein assay reagent, and other analytic-grade chemicals were acquired from Sinopharm Chemical Reagent Co., Ltd. (Beijing, China), unless specified.

### 2.2. Methods

#### 2.2.1. Immobilization of Proteases

Bare silicon dioxide (SiO_2_) microparticles, with a concentration of 100 mg/mL, were dispersed within a coating solution containing PEI, DA, or a combination of both (1:1, *w*/*w*). The final concentration of the coating polymer was 5 mg/mL in a 10 mM Tris-HCl buffer at pH 8. The SiO_2_ microparticles were incubated at 25 °C in an air-bath shaker while being agitated at 150 rpm for 24 h. Following the incubation, the modified SiO_2_ microparticles were recovered using vacuum filtration and subjected to multiple washes with deionized (DI) water to eliminate any remaining polymers.

Based on preliminary tests, both Alcalase and Flavorzyme were immobilized at pH 7.2 (50 mM phosphate buffer) and 25 °C. An amount of 2 g of modified SiO_2_ microparticles was introduced into each 100 mL enzyme solution, with a protein concentration of 0.5 mg/mL. Following a 1 h incubation at 200 rpm, 5 mL of a 1% crosslinking agent (either GA or genipin) was introduced. This led to the establishment of covalent bonds for enzyme immobilization. The impact of extending the incubation period on the immobilization efficiency was investigated. Subsequently, the immobilized enzymes were retrieved via vacuum filtration and subjected to multiple washes with DI water.

The immobilization yield (%) and enzyme loading capacity (mg/g) were determined using the BCA protein concentration kit and calculated using the following equations (Equations (1) and (2)):

(1) $$\mathrm{Immobilization}\;\mathrm{yield}\;(\%)=\frac{C_{0}\times V_{0}-C_{t\;}\times V_{t}}{C_{0}\times V_{0}}$$

(2) $$\mathrm{Loading}\;\mathrm{capacity}\;(\mathrm{mg}/g)=\frac{C_{0}\times V_{0}-C_{t\;}\times V_{t}}{m}$$

*C*_0_ and *C_t_* are the enzyme protein concentration (mg/mL) at the beginning and at the immobilization time *t* (h), respectively, *V*_0_ and *V*_t_ are the volumes (mL) of enzyme solution at beginning and after adding the crosslinking agent, and m (g) is the amount of carrier.

#### 2.2.2. Determination of Enzymatic Activity

The enzymatic activity of both Alcalase and Flavorzyme was measured using a casein substrate according to the method described by Zhu et al. [21] with modifications. Specifically, 400 µL of casein solution (1%, *w*/*w*) was preheated to a defined temperature, and 10 µL of the diluted free Alcalase or Flavorzyme solution was added to start the reaction. For immobilized enzymes, around 10 mg of each sample was used. The reaction was carried out for exactly 10 min and then stopped by adding 20% TCA (*v*/*v*) at a ratio of 1:1 (*v*/*v*) to the reaction system. After centrifuging at 10,000× *g* for 3 min, 200 µL of the supernatant was drawn and mixed with 1000 µL of 500 mM Na_2_CO_3_, as well as with 200 µL Folin and Ciocalteu’s phenol reagent. The mixture was incubated at 40 °C for 20 min, and its absorbance at 680 nm was recorded. The μg of tyrosine equivalents liberated was determined using the standard curve. One activity unit (U/g or U/mL) was defined as at certain pH and temperature, 1 g of immobilized enzyme or 1 mL (g) of initial free enzyme produces 1 µg of tyrosine by hydrolyzing casein in 1 min.

The influence of pH (pH 5~9, 50 mM sodium phosphate buffer; pH 9~12, 50 mM Glycine-NaOH buffer) on the activity of both free and immobilized Alcalase (65 °C) and Flavorzyme (55 °C) was evaluated by adjusting the buffer pH of the casein substrate solution. The effects of temperature on the activity of both free and immobilized proteases were evaluated at pH 9 for Alcalase and at pH 7.5 for Flavorzyme, respectively. The relative enzyme activity of each enzyme at different pH or temperature levels was calculated by setting its activity at their highest level as 100%.

To evaluate the stability of immobilized proteases against pH and temperature stress, these samples were incubated in a buffer with a defined pH at certain temperature for 4 h, and their residual activity was measured. In addition, the reusability of immobilized proteases was evaluated by hydrolyzing soy proteins (pH 9 and 55 °C), and each hydrolysis cycle was around 4 h and the immobilized enzymes were recycled via centrifugation at 3000 g/min. After washing with DI water extensively, the residual activity of recycled immobilized enzymes was measured.

#### 2.2.3. Hydrolysis of Soy Proteins

SPI was dispersed in 100 mL of 50 mM phosphate buffer (5%, *w*/*v*, pH 7.5 or 9) and stirred at 25 °C for 1 h. The SPI dispersion was stored at 4 °C overnight and then its pH was adjusted to the expected value again at 25 °C. After incubating in a water bath at 55 °C for 10 min, it was ready for hydrolysis. Three hydrolysis approaches were carried out and continued for 18 h, i.e., the hydrolysis reaction was started by (1) the addition of free or immobilized Alcalase (E/S, 1000 U/g); (2) adding the mixture of immobilized Alcalase and Flavorzyme (E/S, 500 U/g for each immobilized enzyme); and (3) using immobilized Alcalase (E/S, 1000 U/g) for 30 min at pH 9, after removing the immobilized Alcalase via centrifugation (3000× *g*, 5 min), the immobilized or free Flavorzyme (E/S, 1000 U/g or 10,000 U/g only for its free form) was added at pH 9 or 7.5. Then, 2 mL of each sample was drawn at increasing time intervals and heated to deactivate the enzyme in boiling water for 10 min, and then centrifuged (6000× *g*, 5 min) to obtain the supernatant, or directly separate it from the immobilized enzyme via centrifugation without heating. The supernatant was gathered and stored (−20 °C) for further analysis.

#### 2.2.4. Measurement of Hydrolysis Degree

In total, 100 μL of each hydrolysate sample (or initial SPI dispersion without hydrolysis as the control sample) was mixed with 100 μL 20% TCA solution, and then left to stand for 30 min. After centrifuging at 10,000× *g* for 10 min, 100 μL of the supernatant was taken and diluted appropriately. Then, 100 μL of the diluted solution was mixed with 100 μL of 2 M acetic acid buffer (pH5.4), as well as with 100 μL of ninhydrin color reagent. After thorough mixing, the mixture was boiled for 15 min, followed by cooling in an ice bath. After adding 300 μL of 60% ethanol solution (*v*/*v*), the absorbance of the mixture was measured at 570 nm. The μg of amino group equivalents liberated was determined using the standard curve and the degree of hydrolysis (*DH*) was calculated according to Equation (3):

(3) $$DH=\frac{h}{h_{tot}}=\frac{\left(C_{t}\times D\right)÷C_{0}}{h_{tot}}$$

*h* (meq/g) and *h_tot_* (meq/g) are the equivalent number of hydrolyzed peptide bonds and the equivalent total (average) number of peptide bonds for a given protein (mixture) per g of proteins, *h_tot_* for SPI is 7.8 meq/g [22]; *C_t_* (mmol/L) is the molar centration of liberated amino groups calculated according to the standard curve; D is the dilution factor of the hydrolysate sample; and *C*_0_ is the initial protein concentration (g/L).

#### 2.2.5. Determination of Soluble Protein Content in Hydrolysates

To measure the change in soluble protein content during hydrolysis, each hydrolysate sample was firstly diluted and then centrifuged at 10,000× *g* for 20 min. The protein content in supernatant was determined according to the Kjeldahl method [23] using Kjeltec 8400 (Foss, Hillerød, Denmark), and a nitrogen-to-protein conversion factor of 6.25 was applied.

#### 2.2.6. Peptidomics Analysis of Hydrolysates

The hydrolysates were centrifuged at 10,000× *g* (20 min) and 200 μL of the supernatant was dialyzed against DI water at 4 °C using a dialysis bag with the cut-off of 300 Da for 48 h. The desalted sample was recovered and vacuum-dried completely. After re-dissolving in 1 mL 0.1% formic acid solution, the sample was evenly vortex mixed and centrifuged at 10,000× *g* for 20 min. Then, 200 μL of the supernatant was taken ready for peptidomics analysis.

Nano-HPLC Ultimate 3000 coupled with Q Exactive instrumentation from Thermo Fisher Scientific Inc. (Massachusetts, U.S) was employed for LC-MS/MS analysis. To begin, 1 μL of each sample was introduced into a trap column (Acclaim PepMapTM100; dimensions: 75 μm × 2 cm; material: C18; particle size: 3 μm, pore size: 100 Å) at a flow rate of 3 μL/min. For peptide separation, a capillary column (Acclaim PepMapTMRSLC; dimensions: 75 μm × 15 cm; material: C18; particle size: 2 μm; pore size: 100 Å) was utilized, operating at a flow rate of 300 nL/min. This separation was achieved through a linear gradient of acetonitrile (ACN) and 0.1% formic acid (FA) aqueous solution (*v*/*v*), transitioning from 2% to 40% (*v*/*v*) over the course of 90 min. A data-dependent acquisition (DDA) strategy was implemented in positive mode, featuring a full scan conducted at a resolution of 70,000 within the mass range of 200–3000 *m*/*z*. The top 10 most intense ions were subjected to collision-induced dissociation (CID) for MS/MS scans, carried out at a resolution of 17,500. Two separate hydrolysis samples were prepared, and each of these parallel samples was injected into the LC-MS/MS system three times for analysis. Subsequently, the initial MS data underwent processing using MaxQuant software (version 2.0.1.0) and were searched against the UniProt database (Taxonomy ID 3847, specifically for soy protein). Non-specific cleavages were allowed. A false discovery rate (FDR) of 0.01 was utilized to filter the results. Only peptides identified in all six spectra were considered as present in a given sample. The abundance of each identified peptide across different samples was calculated based on normalized spectral peptide intensity (LFQ intensity).

#### 2.2.7. In Silico Analysis of Identified Peptides

The intensity of bitter taste and the possibility of releasing bioactive peptides after simulating in vitro digestion of each identified peptide were predicted using http://camt.pythonanywhere.com/iBitter-SCM (accessed on 3 March, 2023) [13] and https://biochemia.uwm.edu.pl/biopep-uwm/ (accessed on 18 March, 2023) [24], respectively. These properties of all identified proteins in each sample were collected and visualized according to their relative LFQ intensities in a heatmap (software R studio, version 4.0.0).

#### 2.2.8. Statistical Analysis

Without specification in the above methods, all experiments were performed in triplicate. One-way analysis of variance (ANOVA) was applied to evaluate the significance of differences among mean values at a confidence level of 95%. Tukey’s multiple comparison test was used to compare the significance of differences between each pair of means (*p* ≤ 0.5). A Venn diagram was drawn using the online tool jvenn [25].

## 3. Results

### 3.1. Immobilization of Alcalase and Flavorzyme on SiO_2_ Modified with Different Coating Layers

**Figure 1:**
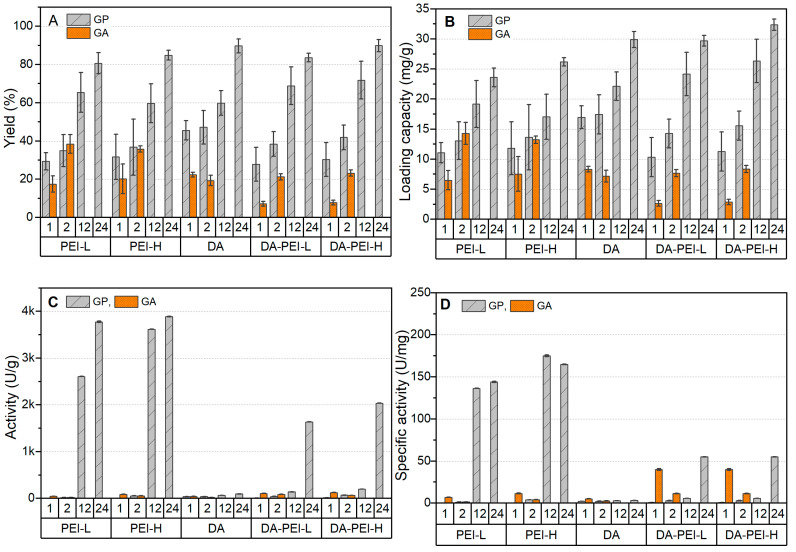
Immobilization efficiency of Alcalase on SiO_2_ microparticles modified with different polymers: (**A**) immobilization yield; (**B**) loading capacity; (**C**) activity; (**D**) specific activity.

**Figure 2:**
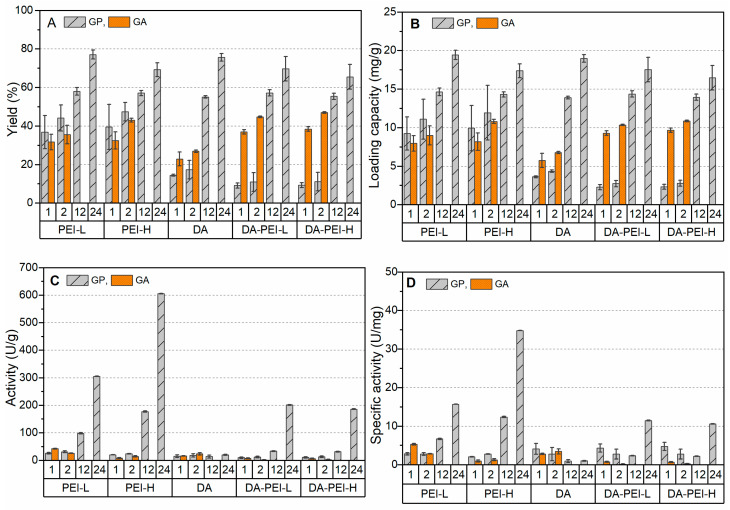
Immobilization efficiency of Flavorzyme on SiO_2_ microparticles modified with different polymers: (**A**) immobilization yield; (**B**) loading capacity; (**C**) activity; (**D**) specific activity.

In this study, both Alcalase and Flavorzyme were individually immobilized on SiO_2_ microparticles modified by PEI-L, PEI-H, DA, DA-PEI-L, and DA-PEI-H, respectively. The crosslinking efficiency of GP and GA were compared over varying reaction periods, specifically up to 2 h for GA and up to 24 h for GP, given that GA is known for its significantly faster crosslinking action compared to GP [19,20]. The results, encompassing both the amounts of immobilized enzymes and the activity of these immobilized enzymes, are presented in Figure 1 and Figure 2 for Alcalase and Flavorzyme, respectively.

As depicted in Figure 1A,B, an extended immobilization period generally contributed to improvements in both the loading capacity and immobilization yield of Alcalase, irrespective of whether GP or GA was applied. Interestingly, GP achieved a comparable immobilized amount of Alcalase in just 2 h, despite the initial expectation of a much slower crosslinking reaction compared to GA. Notably, GP exhibited a higher capacity for enzyme immobilization when DA was introduced to modify the carrier surface. The highest loading capacity for Alcalase was observed on the carrier modified by DA-PEI-H, reaching 31.8 mg/g after 24 h. For carriers modified with PEI-L and PEI-H, the loading amounts reached their maximum values when GP was used for 24 h, specifically 23.5 mg/g and 26.8 mg/g, respectively. In general, when GP was applied for 24 h, the immobilization yield exceeded 80%. However, when GA was used, the loading capacity remained below 15 mg/g on all carriers. This is likely due to the fact that GA primarily induced intra/inter-molecular crosslinking among Alcalase molecules, as evidenced by the extremely low activity observed in all cases when GA was applied (Figure 1C,D). In contrast, the highest activity of immobilized Alcalase using GP reached 3617.8 U/g on SiO_2_-PEI-H, corresponding to a specific activity of approximately 164.0 U/mg protein. Although the presence of DA improved the loading amount of the enzyme, it resulted in a significant decrease in activity.

As shown in Figure 2A,B, the loading capacity of immobilized Flavorzyme steadily increased as the crosslinking time was extended when GP was employed, reaching its peak at approximately 20 mg/g after 24 h. This value was slightly lower than that achieved for Alcalase (Figure 1). There were no significant differences observed in the final immobilization yield among carriers modified by different polymers. However, the presence of DA did slow down the initial immobilization efficiency of Flavorzyme when GP was used. In contrast, when GA was utilized, the immobilization yields of Flavorzyme remained below 50% on all modified carriers, and almost no activity of immobilized Flavorzyme was detected. The highest activity of Flavorzyme was attained at 605.9 U/g (on SiO_2_-PEI-H, using GP), corresponding to a specific activity of approximately 34.8 U/mg protein.

### 3.2. Influence of pH and Temperature on Immobilized Alcalase and Flavorzyme

**Figure 3:**
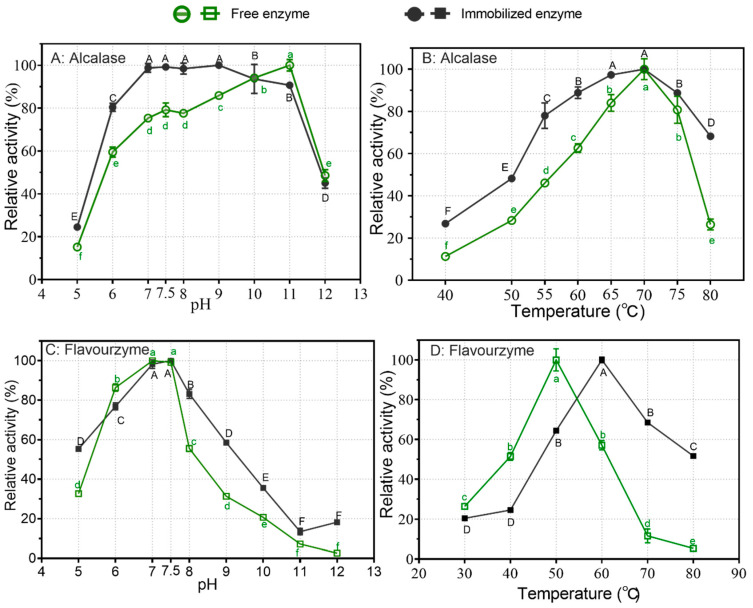
The influence of pH and temperature on the activity of both free and immobilized proteases: (**A**) impact of pH on free and immobilized Alcalase; (**B**) impact of temperature on free and immobilized Alcalase; (**C**) impact of pH on free and immobilized Flavorzyme; (**D**) impact of temperature on free and immobilized Flavorzyme. Different lower-case letters and upper-case letters indicate statistically significant differences in relative activity among different pH or temperature levels for free and immobilized enzymes, respectively (*p* < 0.05).

As per Section 3.1, Alcalase and Flavorzyme were immobilized onto SiO_2_-PEI-H using GP. Subsequently, we characterized the resulting immobilized Alcalase (IA) and immobilized Flavorzyme (IF). The impact of pH on the enzymatic activities of both free and immobilized proteases is illustrated in Figure 3A,C. According to the manufacturer’s recommendations, free Alcalase (FA) exhibited its optimal working pH range between 6.5 and 8.5, despite showing the highest activity against the substrate casein at pH 11 here. However, the optimal pH of IA shifted to pH 7–9. Both free and immobilized Alcalase decreased their activities by 60% at pH 12. On the other hand, immobilized Alcalase (IA) retained 80% of its maximum activity within the broader pH range of 6 to 11, making it suitable for a wider range of industrial applications. Regarding Flavorzyme, both its free and immobilized forms displayed their peak activity at pH 7 and 7.5. Notably, the relative activity of both immobilized Alcalase and Flavorzyme exceeded that of their free forms over a wider pH range. This enhancement may be attributed to the fact that enzyme immobilization, to some extent, stabilizes the enzyme’s spatial conformation, rendering it less susceptible to external factors, including pH variations. Additionally, the coating polymer PEI contains a substantial number of positively charged amino groups, which can effectively neutralize hydroxide ions, resulting in increased stability for immobilized enzymes in alkaline pH environments.

**Figure 4:**
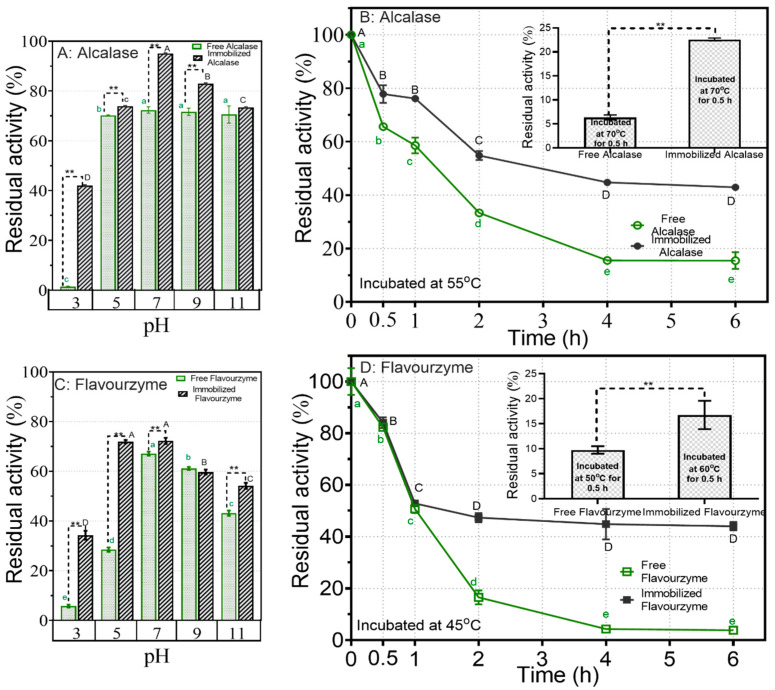
The influence of pH and temperature on the stability of both free and immobilized proteases: (**A**) impact of pH on free and immobilized Alcalase; (**B**) impact of temperature on free and immobilized Alcalase; (**C**) impact of pH on free and immobilized Flavorzyme; (**D**) impact of temperature on free and immobilized Flavorzyme. Different lower-case letters and upper-case letters indicate statistically significant differences in residual activity among different pH or temperature levels for free and immobilized enzymes, respectively (*p* < 0.05), ** indicates a significant statistical difference between free and immobilized enzymes at each pH level (*p* < 0.05).

To assess how pH variations affect enzyme stability, both free and immobilized proteases underwent a 4 h incubation in a buffer with a specific pH at 25 °C. Subsequently, their remaining activity was measured and compared to their initial activity levels. As illustrated in Figure 4A,C, FA retained around 70% activity across the pH range from 5 to 11. Notably, significant increases in retained activity at different pH levels were observed after its immobilization, with nearly 100% activity retention noted at pH 7 after the 4 h incubation period. Compared with FA, free Flavorzyme (FF) exhibited considerably less stability when exposed to pH changes, while immobilization significantly improved its stability across all evaluated pH values. For instance, FF retained only 30% of its activity at pH 5, whereas its immobilized form exhibited more than 70% activity retention. In conclusion, immobilization not only expanded the operational pH range for both proteases but also greatly enhanced their resistance to pH fluctuations.

The effects of temperature on both the activity and stability of immobilized proteases were evaluated at pH 9 for Alcalase and at pH 7.5 for Flavorzyme, respectively. As depicted in Figure 3B, both free and immobilized Alcalase showed the highest activity at 70 °C. Across all the temperature ranges examined, IA consistently maintained higher activity levels than its free counterpart. This difference was particularly noticeable at 80 °C, where free Alcalase exhibited only around 20% of its maximum activity, while immobilized Alcalase retained more than 60% of its peak activity.

Despite both forms of Alcalase displaying their peak activity at 70 °C, their stabilities at this temperature were relatively poor. As illustrated in the insert in Figure 4B, following incubation in a buffer at pH 9 and 70 °C for 30 min, only 5% and 20% of residual activities were observed for FA and IA, respectively. Hence, we also assessed the thermal stability of Alcalase at 55 °C, which is the recommended working temperature according to the manufacturer’s guidelines. In the initial 2 h, both free and immobilized Alcalase showed a rapid decline in activity. Notably, FA retained less than 40% of its activity after 2 h. Immobilization notably enhanced its thermal stability, particularly during prolonged incubation periods. For instance, after 6 h of incubation, IA maintained over 40% of its initial activity.

Regarding Flavorzyme, its optimal temperature shifted from 50 °C to 60 °C following immobilization (Figure 3D). Below 50 °C, FF exhibited higher relative activity compared to its immobilized counterpart, whereas the opposite trend was observed above 50 °C. Specifically, at 80 °C, FF showed almost no activity, while the immobilized form retained approximately 50% of its relative activity. Incubating both free (at 50 °C) and immobilized (at 60 °C) Flavorzyme at their respective optimal temperatures led to significant decreases in activity (Figure 4D). Subsequently, both forms of Flavorzyme were subjected to incubation at 45 °C. During the first hour, both Flavorzymes experienced a 50% reduction in activity. However, following this initial decline, IF remained relatively stable for up to 6 h, whereas its free form nearly lost all of its activity within the same timeframe.

### 3.3. Hydrolysis of Soy Proteins

#### 3.3.1. Hydrolysis of Soy Proteins Using Immobilized and Free Alcalase

The hydrolysis of soy proteins was carried out at pH 7.5 and 9, as these pH values corresponded to the peak activity of IA, as shown in Figure 3A (Section 3.2). Additionally, it is worth noting that peptides derived from soy proteins have been known to readily form either peptide or protein–peptide aggregates, a tendency that is further exacerbated by heat-induced enzyme inactivation [7]. The application of IA can help prevent enzyme inactivation, potentially reducing the formation of peptide aggregates. Consequently, we measured the solubility and degree of hydrolysis (DH) of the hydrolysates generated using IA, both with and without heat treatment, to investigate this problem.

**Figure 5:**
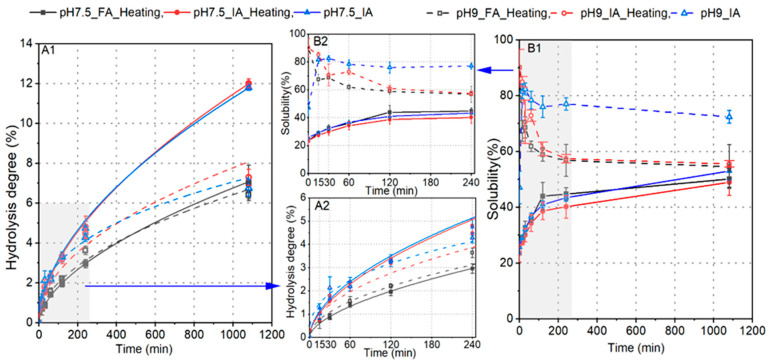
Hydrolysis of soy proteins by FA or IA at pH 7.5 or pH 9: (**A1**,**A2**) increases in DH; (**B1**,**B2**) changes in solubility of hydrolysates.

As shown in Figure 5A, at pH 7.5 the DH values reached approximately 12% after 18 h when using IA, notably surpassing the DH achieved with FA (7.0%). This difference can likely be partly attributed to the fact that the practical activity of FA was 20% lower than its maximum activity, while the addition amounts of both free and immobilized enzymes were calculated based on their respective maximum activities. Upon increasing the hydrolysis pH to 9, the DH values decreased to around 8% with IA, while they still remained slightly higher than that achieved with FA. However, it is important to note that the initial solubility of soy proteins at pH 9 was significantly higher than that at pH 7.5, particularly in cases involving thermal inactivation (boiling water for 10 min), where solubility reached approximately 90.1% (Figure 5B1,B2). During the hydrolysis using either free or immobilized Alcalase at pH 9, the total soluble hydrolysates in supernatants decreased to around 60% in 2 h and remained relatively constant when a thermal inactivation step was included. Contrarily, without thermal treatment, the initial solubility of SPI at pH 9 was 47.1% and it increased to 81.5% in just 15 min during the hydrolysis conducted with IA. Following this hydrolysis, a slight decrease in solubility was observed, while it still maintained a solubility level higher than 70% after 18 h. At pH 7.5, the initial solubility of SPI was only 23.6%, and this was not improved by thermal treatment either. Following hydrolysis, the solubility increased slowly in all cases, yet it remained below 50% after 18 h of hydrolysis.

**Figure 6:**
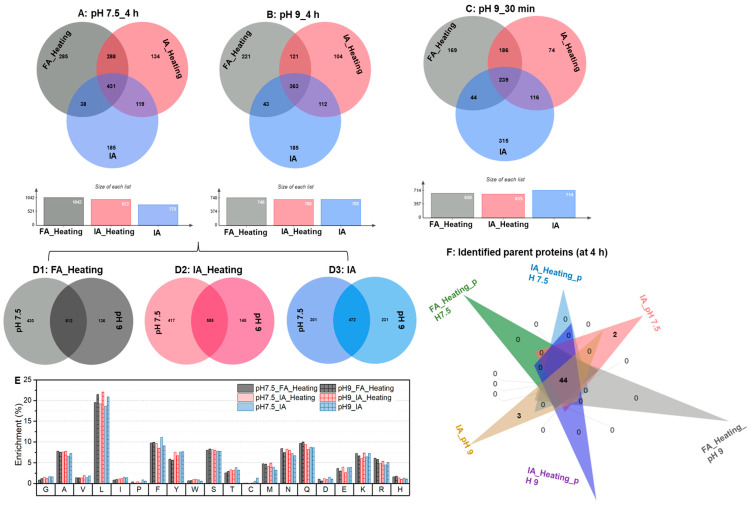
Peptide profiles generated during the hydrolysis of soy proteins by FA or IA: (**A**) hydrolysis at pH 7.5 for 4 h; (**B**) hydrolysis at pH 9 for 4 h; (**C**) hydrolysis at pH 9 for 30 min; (**D1**–**D3**) A comparison of peptide profiles at pH 7.5 and pH 9 generated by the same enzyme preparation at 4 h; (**E**) enrichment of cleavage sites hydrolyzed by FA or IA; (**F**) the leading razar parent proteins suggested for peptides.

The peptide compositions in the soluble fractions of hydrolysates generated at pH 7.5 and pH 9 were determined using LC-MS/MS. As depicted in Figure 6A,B, following a 4 h hydrolysis period, we identified 1042 and 748 peptides in the hydrolysates produced by FA at pH 7.5 and 9, respectively. Out of these, 431 (at pH 7.5) and 363 (at pH 9) peptides were also present in the hydrolysates generated by IA, accounting for approximately 30% of the total identified peptides. Furthermore, when comparing the hydrolysates produced by IA, one with additional thermal treatment, we observed only around 50% overlap in the peptide compositions. There are several factors to consider here. Firstly, the thermal treatment applied to the SPI substrate could induce structural changes, especially at pH 9, which is supported by the significant increase in solubility (see Figure 5B). These structural changes may impact the subsequent release of peptides. On the other hand, Alcalase is known as a non-specific protease, tending to cleave peptide bonds adjacent to hydrophobic amino acids. Hence, it might be challenging to precisely replicate the same peptides across different batches, a topic we will delve into further in Section 3.5. Despite a decrease in the weights of soluble hydrolysates (peptides) after thermal treatment, particularly at pH 9 (as shown in Figure 5B), the number of identified peptides did not undergo significant changes, and even increased at pH 7.5. Interestingly, using FA, a wider array of peptide types was discovered. When employing the same enzyme preparation and inactivation procedure, even though we observed distinct DH and solubility at pH 7.5 and pH 9 (Figure 5), these hydrolysates generally shared 50% of their peptides in common (Figure 6D1–D3). Additionally, we examined the peptide compositions in samples generated at the 30 min mark, during which the hydrolysates from IA exhibited the highest solubility. In these samples, only 239 peptides were found to be in common (as shown in Figure 6C). Despite the variation in peptide profiles across different hydrolysates, the cleavage sites were enriched at the peptide bonds after amino acids L, F, Q, A, S, N, K, and R (Figure 6E), regardless of the enzyme preparation used or the pH conditions applied. Notably, the position after L represented approximately 20% of the peptide bonds. In addition, it is worth noting that a majority of these peptides were likely derived from 44 proteins (Figure 6F), whereas our previous study [26] identified more than 200 proteins in SPI.

#### 3.3.2. Hydrolysis of Soy Proteins Using Immobilized Alcalase and Immobilized Flavorzyme

As previously mentioned, Flavorzyme, which contains exo-peptidases, is anticipated to efficiently break down the hydrophobic amino acids at the N-termini of peptides generated during hydrolysis by Alcalase. This action aims to diminish the potential bitter taste in soy protein hydrolysates [12]. Consequently, Alcalase and Flavorzyme can be employed either in a synchronized or cascade mode. Since IF demonstrated its optimal pH at approximately 7.5, while a notably higher solubility of hydrolysates was observed at pH 9 (Figure 5), we assessed the hydrolysis process at both pH 7.5 and 9.

**Figure 7:**
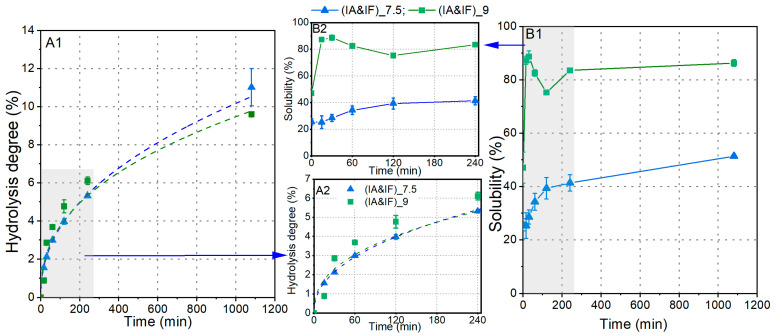
Synchronous hydrolysis of soy proteins using IA and IF together at pH 7.5 and pH 9, respectively: (**A1**,**A2**) increases in DH; (**B1**,**B2**) changes in solubility of hydrolysates.

As depicted in Figure 7A1,A2, the hydrolysis process conducted by IA and IF at both pH 7.5 and 9 resulted in a final DH of approximately 10%, with no significant difference between them. This outcome contrasts with that of the hydrolysis solely by IA (as shown in Figure 5). Furthermore, we also performed hydrolysis of soy proteins using only IF at pH 7.5, but the DH reached less than 2% after 18 h, suggesting that Alcalase was significantly more effective than Flavorzyme in breaking down soy proteins. Despite similar DH values at pH 9 and 7.5, their solubilities exhibited substantial differences. At pH 9, the solubility increased rapidly from 47.1% to 87.3% within the first 15 min, whereas the overall solubility of the hydrolysates generated at pH 7.5 remained below 40%. Additionally, the slight decrease in solubility at pH 9 may have been caused by the aggregation of certain hydrophobic peptides, resulting in their precipitation as insoluble aggregates. Comparing this with the hydrolysis solely by IA at pH 9 (as illustrated in Figure 5), substituting half of the enzyme activity with IF led to slight improvements in both DH and solubility. This suggests that IF may reduce the hydrophobic properties of specific peptides, thus enhancing the measured DH and solubility values.

**Figure 8:**
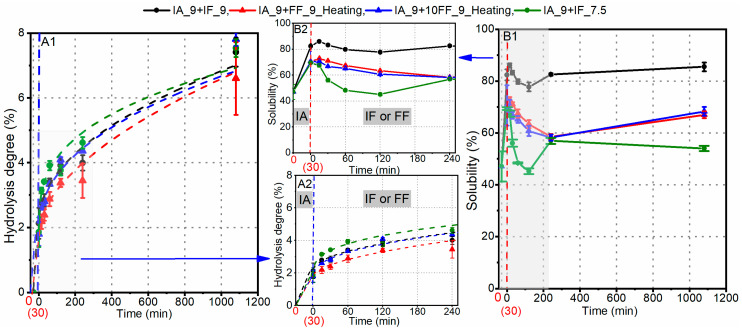
Cascade hydrolysis of soy proteins using IA and FF/IF in a sequence at pH 7.5 and pH 9, respectively: (**A1**,**A2**) increases in DH; (**B1**,**B2**) changes in solubility of hydrolysates.

Differently to the synchronous hydrolysis mode, the cascade hydrolysis process involved initially applying IA for 30 min to maximize the solubility of the hydrolysates, as indicated in Figure 5. Subsequently, IF or FF was introduced to the hydrolysates without IA. Since the optimal pH for IF was approximately 7.5 (Figure 3), IF-mediated hydrolysis was also carried out at pH 7.5. As illustrated in Figure 8, following 30 min of hydrolysis by IA at pH 9, the DH reached approximately 2.1% and the solubility was around 82.4%. Adjusting the pH to 7.5 (IA_9+IF_7.5) led to a 13% decrease in the solubility of these obtained hydrolysates, and further thermal treatment (for the samples IA_9+FF_9_heating and IA_9+10FF_9_heating) reduced the solubility to 70.6%. These results suggested that the peptides generated by IA exhibited much lower solubility at pH 7.5 compared to pH 9. The subsequent hydrolysis by IF at pH 7.5 displayed a slightly faster increase in DH, with DH values reaching approximately 7.4% after 18 h. Additionally, the solubility during the hydrolysis by IF at pH 7.5 decreased more significantly, especially within the first hour. When FF was used instead of IF at pH 9, even increasing its activity tenfold did not enhance the DH, and the solubility decreased notably. Therefore, hydrolysis at pH 9 could maximize the amount of soluble hydrolysates for both the synchronized and cascade hydrolysis modes.

**Figure 9:**
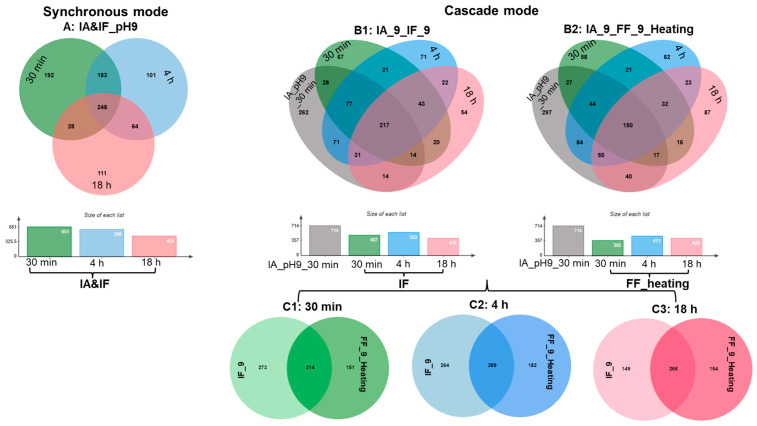
Peptide profiles generated during the hydrolysis of soy proteins using IA and FF/IF: (**A**) Peptide profiles in the hydrolysates generated at pH 9 with a combination of IA and IF for 30 min, 4 h and 18 h; (**B1,B2**) Peptide profiles in the hydrolysates generated at pH 9 firstly with IA for 30 min and then with IF, FF at pH 9, respectivelyfor 30 min, 4 h and 18 h; (**C1,C2,C3**) Peptide profiles in the hydrolysates generated at pH 9 firstly with IA for 30 min and then with IF or FF at pH 9 for 30 min, 4 h and 18 h, respectively.

Following hydrolysis at pH 9, we checked their peptide profiles at 30 min, 4 h, and 18 h. In all cases, the lowest number of identified peptides in the samples were hydrolyzed after 18 h. In cascade hydrolysis mode, in total 714 peptides were found after the initial hydrolysis by IA for 30 min. As the hydrolysis continued with IF or FF, it was observed that their respective hydrolysates shared approximately 33.5%, 39.3%, and 46.7% of peptides in common at 30 min, 4 h, and 18 h, respectively (Figure 9C1–C3). This suggests a convergence towards a more similar peptide profile as the hydrolysis approached its completion, regardless of whether free or immobilized Flavorzyme was employed.

### 3.4. In Silico Analysis of Hydrolysates

**Figure 10:**
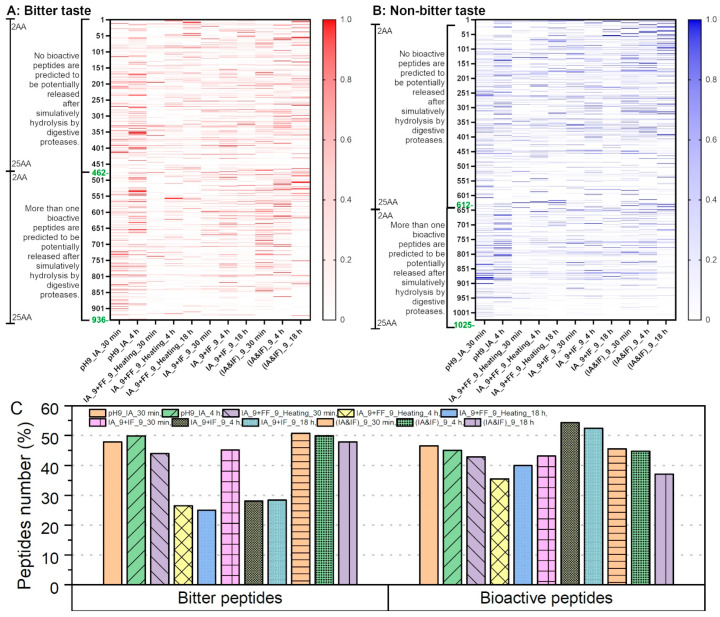
Prediction of bitter taste of identified peptides in different hydrolysates and their potential bioactivity after in silico gastrointestinal hydrolysis using digestive proteases: (**A**,**B**) relative abundance of each peptide using LFQ intensity: (**C**) percentages of bitter peptides and bioactive peptides (after simulative digestion) in different hydrolysates.

In summary, a total of 1961 peptides were identified in various samples, with 936 of these peptides being predicted to possess a bitter taste (Figure 10A). All the identified peptides underwent in silico gastrointestinal digestion, and the bioactivity of the resulting fragments was predicted using the BIOPEP-UWM^TM^ database [24]. Additionally, we calculated the relative abundance of each peptide using LFQ intensity and visualized these data in the form of a heatmap.

As depicted in Figure 10A,B, the initial 30 min hydrolysis using IA resulted in the release of a substantial number of peptides with longer amino acid sequences from soy proteins. Continuing the hydrolysis for 4 h with IA or substituting IA with FF or IF both contributed to the generation of shorter peptides. When compared to these cascade hydrolysis methods, the synchronized hydrolysis using a combination of IA and IF yielded the highest quantity of short peptides after 18 h. However, it is worth noting that the synchronized mode did not effectively reduce the proportion of bitter peptides, as 47.9% of the identified peptides in (IA&IF)_9_18 h were predicted to have a bitter taste (Figure 10C). In contrast, in the hydrolysates produced via the cascade hydrolysis mode using IA and FF/IF after 4 h, less than 30% of the peptides were categorized as bitter peptides. Furthermore, as shown in Figure 10C, over 50% of the peptides in IA_9_IF_4 h and IA_9_IF_18 h were predicted to release bioactive fragments upon simulated digestion by digestive proteases, such as displaying antioxidant activity or ACE inhibition, among others. This percentage was notably higher than that observed in the hydrolysates produced using FF.

### 3.5. Reusability of Immobilized Proteases and Their Reproducibility in Hydrolyzing SPI

**Figure 11:**
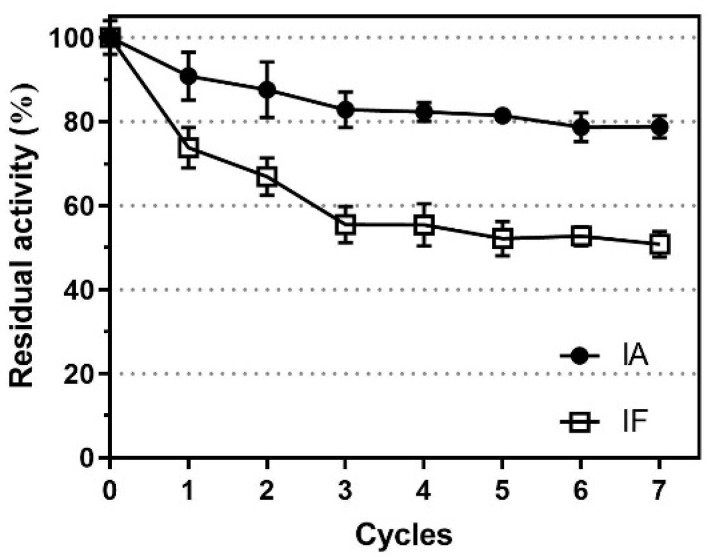
Reusability of IA and IF in the hydrolysis of SPI.

IA and IF were employed to hydrolyze SPI for 4 h at pH 9 and 55 °C, respectively, and their residual activity was measured after each cycle. As illustrated in Figure 11, IA retained approximately 80% of its initial activity after seven cycles. In contrast, the activity of IA incubated in PB buffer (pH 9, 55 °C) declined to only about 40% after 4 h (Figure 4B in Section 3.2). This clearly indicates that in the presence of the substrate (SPI), the immobilized proteases exhibited significantly greater thermal stability. A similar trend was observed for IF as well, as it retained 50% of its initial activity after seven cycles, which was considerably more stable than when it was incubated in PB buffer (Figure 4D in Section 3.2). In addition, these immobilized proteases were air-dried at room temperature and stored at 4 °C for a duration of up to 12 weeks, during which their activities remained nearly constant.

**Figure 12:**
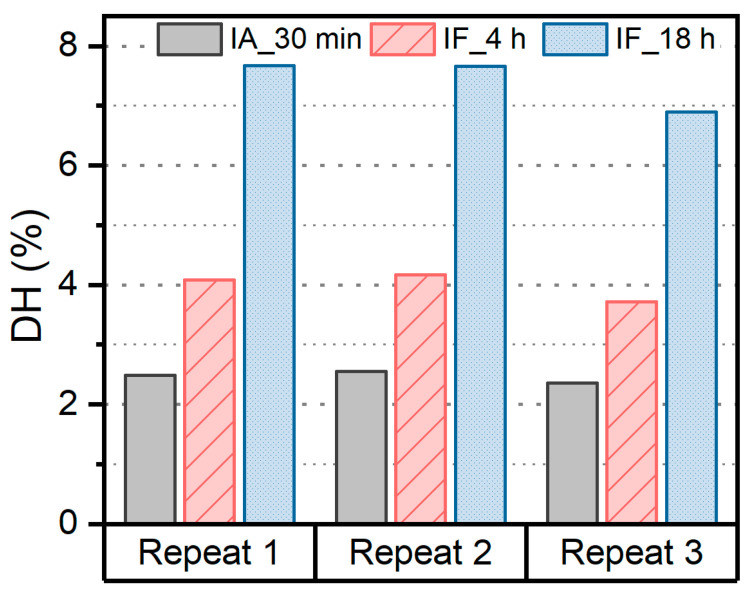
Reproducibility of DH during the hydrolysis of soy proteins using IA or IF.

**Figure 13:**
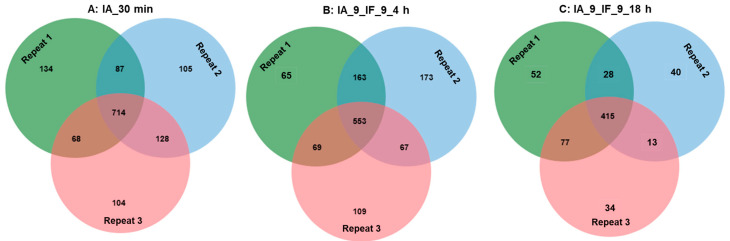
Reproducibility of peptide profile during the hydrolysis of soy proteins using IA (**A**) or the combination of IA and IF (**B**,**C**).

In addition to reusability, we assessed the reproducibility of hydrolysates when the same activity units were added for each repetition, considering both the DH and peptide profiles. As illustrated in Figure 12, the DH exhibited variations of 3.8%, 5.8%, and 5.9% for the hydrolysis using IA_30 min, IF_4 h, and IF_18 h, respectively, indicating a relatively good reproducibility in DH. However, when we examined the peptide profiles, only 53.2% of the peptides overlapped in the hydrolysates produced using IA for 30 min across three repetitions (Figure 13A). Subsequent hydrolysis with IF for 4 h resulted in only 46.1% of peptides being present in all three repetitions. When we extended the hydrolysis to 18 h, the fraction of reproducible peptides increased to 63.0%. Thus, achieving complete hydrolysis contributes to improving the reproducibility of the peptide profile, especially when non-specific proteases are applied, such as Alcalase and Flavorzyme in this study.

## 4. Discussion

The immobilization of enzymes has often resulted in a decline in enzyme activity, partially attributed to limitations in the diffusion of large substrate molecules within the pores where enzyme molecules locate [6,27]. Non-porous SiO_2_ microparticles were opted for as the support. This choice involved a trade-off, sacrificing surface area for enzyme immobilization while circumventing the constraints related to the mass transfer of substrates due to pore diffusion. This aspect is particularly crucial for the hydrolysis of plant proteins, which are typically characterized by low solubility, making it extremely challenging for these substrate molecules to diffuse into pores. By applying a coating layer using PEI, DA, or a combination of both, an enzyme loading capacity of approximately 25 mg/g of support was achieved. This loading capacity is comparable to what is achieved with enzyme immobilization on certain mesoporous microparticles or nanoparticles. Ferreira et al. covalently immobilized Alcalase onto silica derivatives with varying pore sizes (130 nm and 55 nm) and achieved loading capacities of only 2.4–6.3 mg/g of support [28]. In contrast, Zhu et al. immobilized Alcalase on hollow mesoporous silica spheres through metal–protein interaction forces and achieved excellent enzyme loading capacities ranging from 33.7 to 119.3 mg/g [21]. The relatively comparable enzyme loading achieved on these nonporous microparticles can be attributed to the introduction of coating layers. These coatings create a larger surface area for enzyme immobilization compared to bare SiO_2_ particles. This expanded surface area allows for the attachment or adsorption of a greater number of enzyme molecules, thereby increasing the enzyme loading. In comparison to particles modified with DA or a mixture of DA and PEI, those modified with PEI with high molecular weight performed the best in retaining the enzymatic activity of immobilized proteases. Specific activity levels reached approximately 180 U/mg for IA and 35 U/mg for IF. PEI is a branched polymer with amino groups and positive charge, while DA is a catecholamine compound that can form strong adhesive bonds with various surfaces, offering hydroxyl groups while much fewer amino groups than PEI. As previously mentioned, GA, due to its small molecule size, likely triggered intramolecular crosslinking, resulting in a significant reduction in the activity of Alcalase and Flavorzyme during immobilization. On the other hand, GP is believed to crosslink protein molecules by first reacting with the amino groups of the protein or the polymer and then forming dimers between two GP molecules [20]. Therefore, for this one-pot immobilization process, GP proved to be more effective on the support modified with PEI. What is most noteworthy is that this SiO_2_ support is cost-effective, and the one-pot immobilization process can be readily scaled up, making it highly promising for industrial applications.

Regarding the characteristics of immobilized proteases, both IA and IF demonstrated enhanced activity over a wider pH range. This improvement can be attributed to the mitigation of autolysis and denaturation effects, as well as the effective stabilization of enzyme molecules in their immobilized state. Additionally, the proximity of acceptor groups on the carrier surface allowed for control or adjustment of the pH in the microenvironment surrounding the immobilized enzymes as needed. Similar findings were reported by Zhu et al. [21] for the immobilization of Alcalase using a metal affinity approach.

During thermal incubation in a defined buffer, both IA and IF initially exhibited a rapid decline in residual activity but remained quite stable afterward. In contrast, the activity of their free forms continued to decrease throughout the entire incubation period. This improved thermal stability reflects the conformational rigidity of immobilized enzymes, making them resistant to denaturation and autocatalysis. Immobilizing proteases via multiple covalent bonds contributes to stabilizing the enzyme over a broader temperature range, likely due to the emergence of multiple interactions between enzyme molecules and the support material [29]. Here, the initial rapid decline in activity observed in IA and IF during incubation might be attributed to the presence of immobilized enzyme molecules linked by relatively fewer or even single covalent bonds. Remarkably, despite both IA and IF losing about 50% of their initial activity during thermal incubation in the buffer, they exhibited impressive reusability. After undergoing seven cycles of soy protein hydrolysis, IA retained 80% of its initial activity, while IF retained 50%. Likewise, a previous study [30] found that free proteases exhibited significantly improved stability when incubated with a substrate, and this positive effect increased progressively with the extent of hydrolysis (under reactive conditions), suggesting that smaller proteins/peptides even had a greater stabilizing effect for this enzyme.

Lastly, these immobilized proteases were employed in a synergistic process to hydrolyze soy proteins. Soy proteins, a common mixture of plant proteins, are recognized for their capability to produce diverse bioactive peptides. However, the hydrolysis of soy proteins led to a notable formation of insoluble peptide aggregates, reaching up to 40% of the total weight [7]. In this study, the overall solubility of SPI during hydrolysis exhibited a slight increase at pH 7.5 but decreased by approximately 30% at pH 9 when the hydrolysates underwent thermal inactivation. Consequently, it is highly likely that a substantial amount of peptide aggregates were present in this study, and this aggregation was notably exacerbated by heat treatment. Apart from the impact of thermal inactivation, the hydrolysates generated by IA at pH 9 demonstrated significantly higher solubility than those at pH 7.5. An analysis of peptidomics revealed that the majority of peptides were derived from 44 parent proteins, regardless of whether FA or IA was applied. However, the peptide profiles exhibited considerable variations, not only among different hydrolysis approaches but also within repeated batches. This variability is attributed to the nonspecific nature of Alcalase and Flavorzyme, both of which consist of multiple proteases capable of cleaving peptide bonds after a sequence of amino acids, especially hydrophobic ones [11,31]. Consequently, controlling the hydrolysis process becomes a formidable challenge. In practice, industries and researchers typically monitor or terminate the hydrolysis process by measuring the DH. However, this study clearly demonstrated that peptide profiles could vary significantly even at the same DH. In reality, the bioactivity or functionality of protein hydrolysates is determined by their peptide composition rather than DH. Therefore, obtaining hydrolysates with a highly reproducible peptide profile is critical. Achieving complete hydrolysis plays a pivotal role in enhancing the reproducibility of the peptide profile.

Lastly, it is worth noting that the hydrolysis of soy proteins often leads to a bitter taste issue [32]. In the hydrolysates produced through the cascade hydrolysis mode using IA and FF/IF over a 4 h period, less than 30% of the peptides were categorized as bitter peptides. This percentage is significantly lower than that observed in hydrolysates generated by IA alone or in a synchronized hydrolysis involving IA and IF. Considering that bioactive peptides derived from food proteins are typically consumed as part of a diet, it becomes less meaningful to solely assess the bioactivity of individual identified peptides. Instead, it is more pertinent to focus on the bioactivity of the fractions released from these peptides during digestion. In this study, over 50% of the peptides in IA_9_IF_4 h and IA_9_IF_18 h were predicted to release bioactive fragments upon simulated digestion by digestive proteases. Generally, bioinformatics and peptidomics approaches offer a cost-effective and efficient means of predicting, profiling, and screening bioactive soy protein hydrolysates.

## 5. Conclusions

In summary, this study on enzyme immobilization with non-porous SiO_2_ microparticles and the coating layer PEI has shown promising outcomes. This approach to some extent balanced surface area and mass transfer constraints, making this approach a cost-effective and scalable choice for industrial applications. The immobilized proteases, IA and IF, exhibited enhanced activity over a wider pH range, with improved thermal stability and reusability, making them valuable for practical use. The application of IA and IF in soy protein hydrolysis avoided the thermal inactivation step and alleviated insoluble peptide aggregate issues, increasing solubility. Additionally, the analysis of bioinformatics and peptidomics offers an efficient way to understand the functionality of protein hydrolysates. Overall, this study offers practical solutions to enzyme immobilization challenges, with substantial potential for diverse industrial applications.

## Data Availability

The data presented in this study are available on request from the corresponding author.
